# Differences in salient beliefs associated with voluntary exercise training among South Korean firefighters before and after COVID-19

**DOI:** 10.1186/s12889-022-13765-x

**Published:** 2022-07-14

**Authors:** Junhye Kwon, Joohee Choi, Juhyuk Kwon, Chung Gun Lee, Dong-il Seo, Wook Song, Jung-jun Park, Han-joon Lee, Hyun Joo Kang, Yeon Soon Ahn

**Affiliations:** 1 Department of Physical Education, College of Education, Seoul National University, 71-1, 08826 Seoul, South Korea; 2 Institute of Sport Science, Seoul National University, 1 Gwanak-ro, Gwanak-gu, 08826 Seoul, South Korea; 3 Department of Sport Science, College of Liberal Arts, Dongguk University, Gyeongsangbuk-do, 38066 Gyeongju, South Korea; 4 Institute On Aging, Seoul National University, Seoul, 08826 South Korea; 5 School of Sport Science, Pusan National University, Busan, 46241 South Korea; 6 School of Sport Science, University of Ulsan, Ulsan, 44610 South Korea; 7 Department of Sport Medicine, College of Natural Science, Soonchunhyang University, Chungcheongnam-do, Asan, 31538 South Korea; 8 Department of Preventive Medicine and Genomic Cohort Institute, Yonsei Wonju College of Medicine, Yonsei University, Gangwon-do, Wonju, 26493 South Korea

**Keywords:** Theory of planned behavior, Firefighters, Salient beliefs, Elicitation study, Exercise training, COVID-19

## Abstract

**Background:**

Participating in voluntary exercise training is important to meet occupational requirements as well as firefighters’ health and safety. The purpose of this study is to identify salient beliefs associated with voluntary exercise training among firefighters in the pandemic era by comparing outcomes with those from a previous elicitation study, which was carried out before the COVID-19 outbreak.

**Methods:**

A total of 57 firefighters are recruited to participate in an elicitation study. Participants are requested to respond to six open-ended questions related to voluntary exercise training. Content analysis is used to create categories that combine similar factors in each belief. Beliefs mentioned by more than 30% of participants are used for comparison with the results of the previous research.

**Results:**

“Improves my physical ability” (*n* = 44) and “cause injury” (*n* = 17) are identified as behavioral beliefs in the present study, whereas “makes me tired” and “takes too much time” were also elicited in Lee’s study. Normative beliefs are “family members” (*n* = 45) and “colleagues” (*n* = 27) and these results are consistent with those in Lee’s study. “Lack of time” (*n* = 28), “exercise facilities” (*n* = 19), and “COVID-19” (*n* = 19) are elicited as control beliefs in the present study, whereas “physical condition” (*n* = 21) and “exercise partners” (*n* = 14) were elicited as other control beliefs, and “COVID-19” was not mentioned in Lee’s study.

**Conclusion:**

This study can contribute valuable information about salient beliefs associated with exercise training behavior among firefighters, particularly under pandemic conditions. Future researchers should develop tailored exercise training programs for firefighters based on current elicited beliefs.

## Introduction

Firefighting is a physically demanding occupation in which strenuous activities such as carrying heavy tools, lifting patients, and fighting fires are performed in hazardous environments [1–4]. Due to the characteristics of such duties, firefighters are required to maintain a high level of physical fitness. It is well-known that increased physical fitness is positively associated with improved job performance [5]. However, only 44.97% of Korean firefighters meet the guidelines of more than 150 min of moderate to vigorous physical activity per week, as suggested by the World Health Organization [6]. The Korean firefighters' average maximal aerobic capacity (VO_2_max) is 40.23 ml/min/kg, although a VO_2_max of no less than 42 mL/min/kg is strongly recommended [7]. A physical fitness assessment constitutes only 5% of a promotion evaluation in South Korea [8], and participation in exercise training is voluntary. This might have influenced the low level of participation in exercise training among firefighters who do not meet the physical activity guidelines. Not attaining the proposed level of physical fitness can increase the risk of cardiovascular disease (CVD) [9], one of the most frequent causes of worksite fatalities [10]. Therefore, meeting occupational requirements and providing resources such as health and fitness programs are important to maintain firefighters' health and safety [11]. An appropriate exercise training program is required to increase physical fitness in preparation for the professional demands of firefighting [12]. More importantly, key factors that affect participation in exercise training among firefighters should first be identified based on scientific theory.

According to the theory of planned behavior (TPB), attitudes, subjective norms, and perceived behavioral control correspond to behavioral, normative, and control beliefs [13]. Measuring these beliefs gives insight into the roots of a target behavior [14]. Behavioral beliefs affect attitudes toward behavior, normative beliefs consist of determinants of subjective norms, and control beliefs present the basis for the cognition of behavioral control [15]. It is well-documented that eliciting these salient beliefs is vital to develop more effective health-promoting interventions [16]. This information can help with understanding behavior determinants and identifying the most appropriate potential determinants for intervention [17]. However, the elicitation phase has often received little attention [18], and to our knowledge, limited literature is available that identifies key factors in exercise training, particularly among firefighters. More effort should be made to identify salient beliefs among firefighters to change target behaviors.

As COVID-19 is an infectious disease caused by severe acute respiratory syndrome-related coronavirus 2 (SARS-CoV-2) [19], indoor fitness centers were shut down, and athletic training and sports competitions were suspended in many countries [20]. With the continuous rise in infections, a social distancing policy was implemented in South Korea [21]. Despite these unfavorable conditions, firefighters continued to perform their work [22]. However, the rate of exercise training participation among South Korean firefighters is not high and has become even more limited due to the pandemic. Under COVID-19 restrictions, beliefs about exercise training among firefighters may have changed. Identifying the key beliefs influencing firefighters' exercise training under pandemic situations will help with developing future interventions.

Lee et al. [23] conducted an elicitation study to identify salient beliefs associated with exercise training among South Korean firefighters before the COVID-19 pandemic. Ten salient beliefs (i.e., *behavioral beliefs*: “improves my physical ability,” “makes me tired,” “causes injury,” “takes too much time”; *normative beliefs*: “family members,” “colleagues”; and *control beliefs*: “exercise facilities,” “exercise partners,” “lack of time,” “not in good physical condition”) were identified. After an assessment of the structural equation model, three of the ten salient beliefs (i.e., “improves my physical ability,” “takes too much time,” and “colleagues”) were determined to indirectly affect exercise training behavior through intention. Based on these salient beliefs, a tailored exercise training program was developed and implemented for the participants. The present study will provide crucial information that can be used to develop future exercise training programs for South Korean firefighters in the COVID-19 era by comparing outcomes with those from Lee et al. [23]’s elicitation study, which was conducted before the COVID-19 outbreak.

## Methods

### Participants

An elicitation study was carried out in September 2021 to identify modal salient beliefs related to voluntary exercise training among firefighters. A total of 57 (male = 53, female = 4) firefighters from a fire station where the previous research was conducted and whose main duties are firefighting (*n* = 29), rescue (*n* = 14), and administering first-aid (*n* = 14) were randomly recruited to respond to six open-ended questions. A research administrator trained three research assistants on how to conduct the survey. The research assistants distributed consent forms, and all participants provided written consent before the survey. Participants were also provided with an incentive worth approximately $10 after completing the survey. This study was approved by the Yonsei University Institutional Review Board (IRB No. CR318031).

### Interview

Following the procedures of an elicitation study suggested by Fishbein and Ajzen [24], semi-structured interviews asking two questions related to each belief (behavioral, normative, and control) were conducted (presented in Table 1). As confirmed COVID-19 cases kept rising at the time of the survey, face-to-face surveys were considered risky and self-administered online interviews were conducted using Google Forms. The participants were requested to indicate the advantages and disadvantages of participating in exercise training. The participants were also asked to respond to any individuals or groups who would approve or disapprove of their participation in exercise training. Finally, the participants were instructed to list factors that impede or facilitate their exercise training.

**Table 1:** Six open-ended questions used in the present study

Beliefs	Questions
BB	1. What do you think would be the advantages for your participation in exercise training?
	2. What do you think would be the disadvantages for your participation in exercise training?
NB	3. Are there any individuals or groups who would approve of your participation in exercise training?
	4. Are there any individuals or groups who would disapprove of your participation in exercise training?
CB	5. What do you think would make it impede for you to participate in exercise training?
	6. What do you think would make it facilitate for you to participate in exercise training?

*BB* Behavioral beliefs, *NB* Normative beliefs, *CB* Control beliefs

### Content analysis

Content analysis was conducted to create categories that combine similar factors in each belief. As Fishbein and Ajzen recommended, frequently mentioned factors were deemed separate beliefs, whereas factors mentioned by few participants were grouped together [25]. A research administrator and one assistant sorted the responses, and beliefs mentioned by more than 30% (*n* = 17) of the firefighters were used for comparison with the results of the previous research. Coding procedures were applied, and answers pertinent to each specific construct were identified [16]. For the questions on behavioral beliefs, outcomes or consequences of the behavior were identified. For the questions on normative beliefs, the types of individuals or social groups who act as social referents were identified. For the questions on control beliefs, the antecedents or circumstances of the behavior were identified.

## Results

The present study elicited factors of each behavioral, normative, and control belief associated with participation in exercise training among South Korean firefighters. As shown in Table 2, the advantage and disadvantage of exercise training that were mentioned by more than 17 participants in the current study were “improves my physical ability” (*n* = 44) and “cause injury” (*n* = 17), respectively. Meanwhile, “makes me tired” (*n* = 18) and “takes too much time” (*n* = 17) were identified as other behavioral beliefs in Lee et al.’s study (presented in Table 3) [23]. Approving and disapproving referents regarding exercise training who were mentioned by more than 30% of the participants were “family members” (*n* = 45) and “colleagues” (*n* = 27) (see Table 2). These beliefs (*n* = 43 and *n* = 34, respectively) were consistent with the results of Lee et al. [23] in terms of normative referents (shown in Table 3). Finally, facilitating or impeding factors that were indicated by more than 30% of the firefighters were “lack of time” (*n* = 28), “exercise facilities” (*n* = 19), and “COVID-19” (*n* = 19). “Physical condition” (*n* = 21) and “exercise partners” (*n* = 14) were elicited as other control beliefs, and “COVID-19” was not mentioned by Lee et al. [23] (shown in Tables 2 and 3).

**Table 2:** Behavioral, normative, and control beliefs elicited by firefighters regarding participating in exercise training (*n* = 57)

Beliefs	Factors	*n*
Behavioral beliefs	**Improves my physical ability**	**44**
	**Causes injury**	**17**
	Makes me tired	13
	Self-confidence	13
	Have positive mindset	8
	Relieve stress	7
	Takes too much time	7
	Have exercise compulsion / addiction	4
	Feel accomplishment	3
Normative beliefs	**Family members**	**45**
	**Colleagues**	**27**
	Friends	16
	Government / Fire department	3
	Exercise circles	2
	Civilians	1
	Doctors	1
Control Beliefs	**Lack of time**	**28**
	**COVID-19**	**19**
	**Exercise facilities**	**19**
	Characteristics of occupation	16
	Physical condition	13
	Exercise trainer	8
	Accessibility of exercise facilities	6
	Economic determinants	2
	Workplace environment (atmosphere)	2
	How to exercise / YouTube	2
	Habituation	2
	Exercise interest	1
	Exercise partners	1
	Perception about beginner	1

Bolded items are beliefs mentioned by more than 30% of participants

**Table 3:** Behavioral, normative, and control beliefs elicited by firefighters regarding participating in exercise training in Lee’s study (*n* = 29)

Beliefs	Elicited variables	*n*
Behavioral beliefs	Improves my physical ability	30
	Causes injury	22
	Makes me tired	18
	Takes too much time	17
Normative Beliefs	Family members	43
	Colleagues	34
Control Beliefs	Exercise facilities	31
	Physical condition	21
	Exercise partners	14
	Lack of time	11

Beliefs mentioned by more than 30% of participants are presented

## Discussion

To our knowledge, this is the first study to identify salient beliefs associated with participation in exercise training among South Korean firefighters in the COVID-19 era. Although similar results were found between the present and previous studies, some notable differences should be addressed in the discussion. A total of seven beliefs were derived from this study, whereas ten beliefs were elicited from the previous study. Among the behavioral beliefs, firefighters believe that participating in exercise training improves their physical ability. “Improves physical ability” was also the single most frequently identified salient belief among university students [26]. Another behavioral belief identified in our study was “cause injury.” According to the elicitation, firefighters perceive that exercise training may cause injury if they exercise incorrectly or overtrained. “Cause injury” was identified as one of the salient behavioral beliefs of physical activity behavior among young people in a previous study [27] and one of the most commonly mentioned behavioral disadvantages among various populations, including worksite groups, in a previous review [26]. These two factors were also reported as key behavioral beliefs by Lee et al. [23]. Therefore, future studies should develop specific strategies that emphasize that exercise training does not always cause injury.

The rest of the behavioral beliefs identified by Lee et al. [23] were “makes me tired” and “takes too much time.” In our study, however, these two behavioral beliefs were mentioned by only 23% (*n* = 13) and 12% (*n* = 7), respectively, whereas both beliefs were mentioned by more than 30% of the participants according to Lee et al. [23]. Lee and his colleagues developed an exercise training program based on these elicited beliefs and applied several interventions with specific messages (i.e., firefighters can participate in exercise training during standby time between emergency calls) [23]. Therefore, fewer firefighters may have perceived participation in exercise training as a waste of time.

In terms of normative beliefs, firefighters believe that “family members” and “colleagues” think they should participate in exercise training. Downs and Hausenblas found that “family members” have the strongest normative effect on exercise behavior in a diverse population [26]. Likewise, “colleagues” were also one of the most commonly mentioned normative referents for exercise behavior, particularly among worksite populations [26]. These two factors were identified as key normative beliefs related to exercise training by Lee et al. [23] as well. As in other populations, firefighters most value the opinions and thoughts of “family members” and “colleagues” about their behavior, even in this pandemic era. Accordingly, exercise training programs for firefighters should focus more on these referents. Apart from “friends” (*n* = 16), the rest of the normative beliefs in our study were markedly less mentioned.

Similar to the results of Lee et al. [23], firefighters think that “lack of time” is one factor that hinders their participation in exercise training. According to previous work reported by the Korea Labor Institute, nearly 14% of firefighters responded that they lack exercise time because of the heavy workload [28]. The National Fire Agency has established the 24-h “COVID-19 Crisis Response Support Headquarters” as an operating system to support transportation in cooperation with local public health centers since the crisis alert level entered the “orange” stage [29]. Therefore, firefighters may have had much less time to participate in exercise training. On the one hand, 11 firefighters mentioned a “lack of time,” which is approximately 39% of the total participants in Lee et al. [23]. On the other hand, in our study, the “lack of time” was mentioned by 28 firefighters (49%), which may mean that the time issue has become a more important belief that negatively affected participation in exercise training among firefighters. Firefighters could be informed of forms of exercise training that are not time-consuming to address the salience of time; for example, a short exercise training program that can be done in between calls. This would target the control belief of “lack of time” while including exercise training as part of their duties. “Exercise facilities” is another control belief related to difficult or favorable situations for exercising. Firefighters reported that neat and tidy exercise facilities make it easier to participate in exercise training. By contrast, shabby exercise facilities with a lack of equipment hamper their exercise training. “Exercise facilities” was one of the frequently mentioned issues among various groups [26] and firefighters [30]. Undoubtedly, social distancing measures were helpful in reducing COVID-19 cases; however, it has negatively affected exercise behavior due to social meeting restrictions and closures of indoor gyms [31, 32]. More specifically, firefighters in our study commented that they could not use the exercise training room in the fire station due to COVID-19 restrictions. This may have strengthened the salient belief that exercise facilities negatively affect participation in exercise training. One notable result was that “exercise partners” was mentioned by only one firefighter in our study, whereas it was mentioned by 14 firefighters (48%) in Lee et al. [23]. (see Table 3). As mentioned above, the government enforced a social distancing policy and regulated social gatherings, thereby further restricting opportunities to participate in exercise training together [21]. For this reason, most firefighters may believe that “exercise partners” facilitate their exercise training behavior.

Interestingly, “COVID-19” was identified as another control belief that hindered firefighters’ exercise training behavior. According to the interview responses, regarding the COVID-19 factor, the firefighters were not afraid of being infected by the virus. Instead, the COVID-19 measures for indoor sports facilities and those related to gathering size limits impeded the firefighters’ exercise training behavior. COVID-19 measures such as the prohibition of private gatherings to prevent infection, especially for public servants (such as firefighters) [33], might have led the firefighters to think of COVID-19 as an exercise training obstacle. Additionally, “physical condition” was identified as another control belief by Lee et al. [23], whereas it was mentioned by less than 30% of the participants in the current study. According to the Firefighting Science Research Center, 57.2% (*n* = 1934) of 3381 firefighters reported that their workload became a lot heavier after COVID-19 broke out [34]. Likewise, the pandemic situation has been making frontline workers busier. Hence, firefighters may believe that the COVID-19 pandemic has hampered their exercise training more than physical conditions. Firefighters need to maintain a high level of physical fitness to meet occupational demands and for their health and safety. However, firefighters seem to have difficulty exercising due to COVID-19 measures. Health authorities and firefighting officials should consider the salient beliefs related to exercise training behavior when developing future policies and exercise training programs for firefighters in a pandemic situation.

Although the present study has notable findings, there are some limitations. First, this study only proceeded the first step of TPB procedures, and therefore, we could not clarify which salient beliefs indirectly affect exercise training behavior through intention. Future research should conduct the next step to provide more information for the development of appropriate interventions for firefighters. Second, our study may not be applicable to all South Korean firefighters because we only targeted firefighters from a single fire station. Thus, modifications may be needed to apply our findings to other contexts.

## Conclusion

Despite the abovementioned limitations, the results of our study can contribute valuable information about salient beliefs associated with exercise training behavior among firefighters, particularly under pandemic conditions. Future researchers should develop tailored exercise training programs for target populations based on current elicited beliefs. Moreover, compared to the previous research conducted by Lee et al. [23], COVID-19 is a new notable factor that hindered firefighters’ participation in exercise training. Further implementation of exercise training interventions should consider the pandemic situation.

## Data Availability

The datasets generated and/or analysed during the current study are not publicly available due due the terms of consent/assent to which the participants agreed but are available from the corresponding author on reasonable request.

## Abbreviations

CVD: Cardiovascular disease
COVID-19: Coronavirus disease of 2019
SARS-CoV-2: Severe acute respiratory syndrome-related coronavirus 2
TPB: Theory of planned behavior
